# Smoking-Related Knowledge, Attitudes, Behaviors, Smoking Cessation Idea and Education Level among Young Adult Male Smokers in Chongqing, China

**DOI:** 10.3390/ijerph120202135

**Published:** 2015-02-16

**Authors:** Xianglong Xu, Lingli Liu, Manoj Sharma, Yong Zhao

**Affiliations:** 1 School of Public Health and Management, Chongqing Medical University, Chongqing 400016, China; E-Mails: xianglong1989@126.com (X.X.); 13110265315@163.com (L.L.); 2 Research Center for Medicine and Social Development, Chongqing Medical University, Chongqing 400016, China; 3 The Innovation Center for Social Risk Governance in Health, Chongqing Medical University, Chongqing 400016, China; 4 Department of Behavioral and Environmental Health, Jackson State University, Jackson 39213, USA; E-Mail: manoj.sharma@jsums.edu

**Keywords:** smoking cessation, knowledge, attitude, young smokers, practice, male, education level

## Abstract

*Introduction*: In 2012 in China, 52.9% of men were reported to smoke while only 2.4% of women smoked. This study explored the smoking-related Knowledge, Attitudes and Practices (KAP) among young adult male smokers. *Methods*: A cross-sectional study was conducted in four municipal areas of Chongqing using a questionnaire administered to 536 natives young male smokers aged 18–45 years old. *Results*: The total score of smoking cognition, the total score of smoking attitude and the total score of positive behavior to quit smoking was significantly different among the three groups by education. Besides, 30.97% of male smokers never seriously thought about quitting smoking. Logistic regression analysis found smoking-related knowledge, attitudes, behaviors and sociodemographic factors affect having smoking cessation idea. But no statistically significant correlation was observed between smoking cognition and positive behavior to quit smoking in a sample of higher education. No statistically significant correlation was observed between smoking cognition and positive behavior to quit smoking (Pearson correlation coefficient = 0.03012, *p* = 0.6811), and also no statistically significant correlation was observed between smoking cognition and positive behavior to quit smoking (Pearson correlation coefficient = 0.08869, *p* = 0.2364)  in the sample of higher education young adult males *Conclusions*: Young adult males with higher education have a better knowledge of smoking hazards and a more positive attitude toward smoking, however, this knowledge and attitude do not necessarily translate into health behavioral outcomes such as not smoking. Overall the present findings indicate that no statistically significant correlation between the education level and quitting smoking idea exists among young adult male smokers in China. This survey gives a snapshot of the impact of education on smoking-related KAP among young adults male smokers.

## 1. Introduction

Cigarette smoking is the leading cause of preventable diseases and premature death [1], and it is responsible for more than 5 million deaths every year [2]. Smoking can increase the risk of cardiovascular disease, respiratory disease, and 10 different forms of cancer [3,4]. In many low-and middle-income countries, women smoke much less than men. According to the survey, worldwide it was estimated that men smoked nearly five times as much as women [5]. Furthermore in China, in 2012, 52.9% of men were reported to be tobacco smokers while only 2.4% of women smoked [6]. The 2009 Egypt Global Adult Survey also showed that 37.7% men and 0.5% women in Egypt currently smoked tobacco [7]. Female smoking prevalence has been low in China, however male smoking prevalence has been high for several decades. The reason for the difference is generally attributed to strong and persistent social norms against female smoking [8,9]. It is perhaps less well known that female smoking rates have actually declined through most of the 20th century in China. In contrast, male smoking prevalence rate in the 1908–1912 birth cohorts was 70% and the prevalence remained high in later male cohorts [8]. In most countries, males tend to engage more frequently in the most adverse health-related behaviors than females [9,10,11]. 

In recent years, many highly educated individuals (such as doctors, teachers) accounted for a high proportion of smoking in China. A study showed that in China few physicians promote smoking cessation, and the smoking rate among male physicians is above 50% [12]. In 2008, Survey on Smoking-related Knowledge, Attitudes and Behavior of Teachers across China found that 19.6% of teachers smoked [13]. A study showed that the desire and intention were independent predictors of quit attempts [14]. Having quit smoking idea is an important step in the initiation of smoking cessation. To explore the influencing factors of male smokers, quit smoking idea is very important for quit attempts. These characteristics not only directly affect the next steps one takes to quit smoking, but also provide the basis for further health education. Education level was used as a socioeconomic indicator and was selected on the basis of a number of considerations. Previous studies showed that educational level is an important socioeconomic factor in health education and promotion research, as it may influence knowledge and behavior that are important for making health behavior choices, [15,16], for example those concerning smoking. Studies focusing on the entire population found that smoking was significantly associated with education level [17,18], but there is no in-depth study on the association, especially in young adult males. Young adult male are important smoking subpopulation in China. Global Adult Tobacco Survey (GATS) China 2010 Country Report showed that the smoking prevalence for men aged 15 and older in China was 52.9% [19]. However, few research studies assessing the relationship between education level and smoking in a sample of young adult male smokers. The aim of this study was to probe tobacco-related knowledge, attitudes and practices among young adult male smokers and provide evidence for the intervention of smoking cessation. 

## 2. Materials and Methods

### 2.1. Participants and Methods

#### 2.1.1. Ethical Approval 

All subjects gave their informed consent for inclusion before they participated in the study. The study was conducted in accordance with the Declaration of Helsinki, and the protocol was approved by the Ethics Committee of Chongqing Medical University (2011159).

#### 2.1.2. Population and Sample 

In 2002, a survey of smoking and passive smoking in China showed that there was no significant difference on the prevalence of male smoking in different area in China [20]. Therefore, compared to the prevalence of male smoking in other regions in China, there were no major differences in Chongqing. Data were collected through questionnaire surveys. Eligible respondents were persons aged between 18 and 45 years old and resided in Chongqing over 6 months at the time of the survey. Sampling sites included downtown, commercial walking street in four main urban areas of Chongqing. A questionnaire survey administered in June 2011 generated representative data from the four urban areas, namely, Yuzhong District, Shapingba District, Jiulongpo District, Nanan District were selected by computer generated randomization from nine urban districts in Chongqing, China. According to the literature [19], the current smoking prevalence was 28.1% among the total adult population and the percentage of those who were aware that smoking could cause all three diseases (stroke, heart disease and lung cancer) was 23.2%. We set *P* = 0.3 (*P* = 0.3; *Q* = 1 ‒ *P =* 1 ‒0.3 = 0.7 margin of error *d* = 0.10, *P* = 0.10 × 0.30 = 0.03, *Z_α_* = 1.96; sampling size = 897). The actual total sample size for the survey was 1265 18 to 45 year-olds. Among the 1342 interviewees, the response rate was 1323 (98.6%); nine responses were deleted due to missing data, resulting in a final sample of 1265 in the analysis. Finally, the study included 536 male smokers. Current smokers included all patients smoking tobacco on a daily basis. We asked all respondents whether they had smoked cigarettes in their lifetimes. Current smoker was defined as a person who smoked tobacco at the time of the interview. 

### 2.2. Questionnaire Interview

A standardized questionnaire, designed based on tobacco control knowledge-attitude- practice model among young adult male smokers in Chongqing, China, 2011, that covered demographic characteristics—age, educational level, dwelling time (Six months to one year/Above one year) was assessed by self-reported, and smoking-related knowledge-attitude-practice—knowledge and attitude about tobacco-related disease, smoking cessation-related thoughts and practice, past smoking practice, was performed by trained investigators. Education level was categorized as ≤primary school, junior middle school (basic education), ≥a senior high school (including vocational/technical secondary school and junior college), (secondary education) and ≥senior college and university (higher education). Age was categorized as 18–25 years, 26–35 years and 36–45 years. The questionnaire was developed based on the knowledge–attitude–behavior model specially designed for the target population and finalized after repeated discussions with experts to establish face and content validity and the pilot investigation which was done with a group of 30 medical students.

### 2.3. Survey Implementation 

The pilot test was carried out in a medical university and 30 students took the pretest. In June 2011, a formal investigation was conducted in four main urban areas of Chongqing. We adopted non-probability sampling. At each survey locations, participates conformed to inclusion criteria and exclusion criteria which were selected randomly and asked politely if they would like to participate in the campaign, and then who agreed were interviewed face to face by investigators to answer every designed question. The interview time lasted for approximately 10–20 min for each participant.

### 2.4. Data analysis

The questionnaires data were carefully checked before entering into the database using Epi-data 3.1 software. After a meticulous sorting, data cleaning and analyses were performed by statistical analysis system software (version 9.1; SAS Institute, Cary, NC, USA). Missing data were excluded and all data entries were double-checked in order to prevent errors. χ^2^ test was used to compare differences in categorical variables, and ANOVA was used to compare differences in continuous variables in three education levels. All statistics were performed using a 2-sided test and statistical significance was considered at *p* < 0.05. We utilized logistic regression analysis to examine the effect of the advertisement.

## 3. Result

### 3.1. Characteristics of the Sample 

Table 1 displayed descriptive statistics for male smokers. The total of 536 smokers comprised 12.50% with basic education, 53.92% with secondary education and 33.58% with higher education. Significant differences were found with respect to smokers’ age (*p* < 0.0001), the total score of smoking cognition (*p* < 0.0001), and the total score of smoking attitude (*p* < 0.0283) (Table 1).

### 3.2. Knowledge of Smoking-Related Hazards

Table 2 showed different education level smokers’ cognition of smoking’s related hazards. Significant differences in the mean score of cognition that “Smoking causes lung disease (*p* = 0.0008)”, “smoking causes oral cancer (*p* < 0.0001)”, “Smoking causes heart disease (*p* = 0.0172)”, “Smoking causes stroke (*p* = 0.0008)”, and “Smoking causes impotence (*p* = 0.0021)” were observed among three groups. 

**Table 1:** Characteristics of the study participants, Chongqing, China, 2011.

Variables	Basic Education (*n* = 67)	Secondary Education (n = 289)	Higher Education (*n* = 180)	*p*-Value
Age (%)				
18–25 years	28.36	39.10 ^▲^	77.78 ^▲^	<0.0001 **
26–35 years	22.39	32.87	16.11	
36–45 years	49.25 ^▲^	28.03	6.11	
Dwelling time (%)				
Six months to one year	5.97	14.88	15.00	0.1188
Above one year	94.03 ^▲^	85.12 ^▲^	85.00 ^▲^	
The total score of smoking cognition (mean, SD)	4.28 ± 2.56	5.99 ± 2.52	6.11 ± 2.55	<0.0001 **
The total score of smoking attitude (mean, SD)	3.03 ± 1.33	3.63 ± 1.27	3.67 ± 1.15	0.0355 *
The total score of positive behavior to quit smoking (mean, SD)	6.64 ± 2.37	7.21 ± 2.05	7.17 ± 2.79	0.0002 **

Notes: (1) ^▲^ The largest number of options; * statistically significant (*p* < 0.05); ** statistically significant (*p* < 0.001); (2) The total score of smoking cognition is Table 2 all scores addition; (3) The total score of smoking attitude is Table 3 all scores addition; (4) The total score of positive behavior to quit smoking is Table 4 all scores addition, except the amount of smoking Compared with the past 1 month; (5) χ^2^ test was used to compare differences in categorical variables, and ANOVA was used to compare differences in continuous variables; (6) Abbreviations: SD, standard deviation.

**Table 2:** Smokers’ cognition of smoking’s related hazards, Chongqing, China, 2011.

Items	Basic Education (*n* = 67)	Secondary Education (*n* = 289)	Higher Education (*n* = 180)	*p-*Value
*Smoking cause the following (mean, SD)*				
Lung disease	0.85 ± 0.36	0.96 ± 0.18	0.95 ± 0.22	0.0008 *
Oral cancer	0.30 ± 0.46	0.64 ± 0.47	0.55 ± 0.49	<0.0001 *
Heart disease	0.25 ± 0.43	0.43 ± 0.49	0.40 ± 0.49	0.0172 *
Stroke	0.06 ± 0.24	0.19 ± 0.39	0.27 ± 0.44	0.0008 *
Impotence	0.09 ± 0.28	0.25 ± 0.43	0.30 ± 0.46	0.0021 *
*Exposure to second-smoke cause the following (mean, SD)*				
Lung cancer in non-smokers	0.65 ± 0.47	0.77 ± 0.42	0.81 ± 0.39	0.0672
Lung disease in children	0.58 ± 0.49	0.78 ± 0.41	0.80 ± 0.39	0.0006 *
Heart disease	0.26 ± 0.44	0.38 ± 0.48	0.46 ± 0.49	0.0190 *
Birth of low-weight babies when the pregnant mother has been exposed to cigarette smoking (mean, SD)	0.46 ± 0.50	0.70 ± 0.45	0.66 ± 0.47	0.0009 *
Smoking causes serious harm to one’s health (mean, SD)	0.74 ± 0.43	0.84 ± 0.36	0.87 ± 0.32	0.0422 *

Notes: (1) * statistically significant (*p* < 0.05); (2) Every question’s highest score is 1, the lowest score is 0. The higher the score represents the knowledge of smoking is harm to health is better, the beliefs and attitudes are more positive, and the behavior is more conducive to health; (3) The correct cognition is choose “yes” and score of 1, incorrect cognition refers is choose “no” or “do not know”, and score of 0; (4) ANOVA was used to compare differences in continuous variables; (5) Abbreviations: SD, standard deviation.

Considering second-smoke hazards, the differences were significant in the mean score of cognition that “Exposure to smoke from another person’s cigarette cause lung disease in children (*p* = 0.0006)”, “Exposure to smoke from another person’s cigarette cause heart disease and birth of low-weight babies when the pregnant mother has been exposed to cigarette smoking (*p* = 0.0190)”, “Smoking cigarettes cause serious harm to one’s health (*p* = 0.0009)” and “Exposure to smoke from another person’s cigarette causes heart attack (*p* = 0.0422)”. However, there was no statistically significant difference in the mean score of “Exposure to smoke from another person’s cigarette cause lung cancer in non-smokers (*p* = 0.0672)”.

### 3.3. Smoking-Related Attitudes and Beliefs

Among three groups, there were differences in the mean score of attitude and beliefs that think “I am concerned about my health when someone is smoking near me (*p* = 0.0010)”, believe “Exposure to smoke from another person’s cigarette causes heart attack (*p* = 0.0040)”. However, no statistically significant difference in the mean score of “The people around me (including family members, friends, and colleagues) believe that I should not smoke” (*p* = 0.3688), “Quitting smoking would improve my health” (*p* = 0.2448) and “Whether to support the provisions that establish smoking bans in public places/ workplace” (*p* = 0.0719). Information on male smokers’ attitude towards smoking-related hazards is summarized in Table 3.

**Table 3:** Smokers’ smoking-related attitude, Chongqing, China, 2011.

Items	Basic Education (*n* = 67)	Secondary Education (*n* = 289)	Higher Education (*n* = 180)	*p*-Value
I am concerned about my health when someone is smoking near me (mean, SD)	0.47 ± 0.50	0.68 ± 0.46	0.73 ± 0.44	0.0010 *
Believing Exposure to smoke from another person’s cigarette causes heart attack (mean, SD)	0.31 ± 0.46	0.49 ± 0.50	0.54 ± 0.49	0.0040 *
The people around me (including family members, friends, and colleagues) believe that I should not smoke (mean, SD)	0.64 ± 0.48	0.71 ± 0.45	0.733 ± 0.44	0.3688
Quitting smoking would improve my health (mean, SD)	0.71 ± 0.45	0.79 ± 0.40	0.74 ± 0.43	0.2448
Whether to support the provisions that establishment smoking bans in public places/workplace (mean, SD)	0.86 ± 0.34	0.94 ± 0.23	0.90 ± 0.30	0.0719

Note: (1) * statistically significant (*p* < 0.05); (2) Every question’s highest score is 1, the lowest score is 0. The higher the score represents the knowledge of smoking is harm to health is better, the beliefs and attitudes are more positive, and the behavior is more conducive to health; (3) χ^2^ test was used to compare differences in categorical variables, and ANOVA was used to compare differences in continuous variables; (4) Abbreviations: SD, standard deviation.

### 3.4. Smoking-Related Practices

Statistically significant differences were observed as regards thinking smoking will harm themselves (*p* = 0.0002), and considering smoking will harm others (*p* = 0.0001). But there were no statistically significant differences in the majority of smokers on sometimes discussed the relationship between smoking and health at home (71.64%, 77.85% and 74.44%, respectively) and never seriously thought about quitting smoking (34.33%, 30.80% and 30.00%, respectively) (Table 4).

**Table 4:** Smoking-related behavior, Chongqing, China, 2011.

Items	Basic Education (*n* = 67)	Secondary Education (*n* = 289)	Higher Education (*n* = 180)	*p*-Value
*Compared with the past 1 months*				
The amount of smoking (%)				
Hard to say	8.95	13.49	17.22	0.3241
Smoke Less	14.93	23.53	20.56	
Smoke Same	65.67 ^▲^	56.06 ^▲^	53.33 ^▲^	
Smoke More	10.45	6.92	8.89	
*Over the past 1 month, occurrence these things’ frequency*				
To discuss the relationship between smoking and health at home (%)				
Never	26.87	16.61	17.22	0.1050
Sometimes	71.64 ^▲^	77.85 ^▲^	74.44 ^▲^	
Often	1.49	5.54	8.34	
Consider smoking will harm yourself (%)				
Never	38.81	18.34	18.89	0.0002 **
Sometimes	44.78 ^▲^	68.51 ^▲^	59.44 ^▲^	
Often	16.42	13.15	21.67	
Think smoking will harm others (%)				
Never	40.30	25.61	22.78	0.0001 **
Sometimes	43.18 ^▲^	61.59 ^▲^	50.00 ^▲^	
Often	16.42	12.80	27.22	
Seriously thought about quitting smoking (%)				
Never	34.33	30.80	30.00	0.4505
Sometimes	50.75 ^▲^	54.33 ^▲^	48.89 ^▲^	
Often	14.93	14.88	21.11	

Note: (1) ^▲^ The largest number of options; ** statistically significant (*p* < 0.001); (2) χ^2^ test was used to compare differences in categorical variables.

### 3.5. Correlation Analyses of Knowledge, Attitude and Behaviors

A statistically significant correlation was observed between smoking cognition and smoking attitude (Basic education: Pearson’s correlation coefficient = 0.60998, Secondary education: Pearson’s correlation coefficient = 0.54064, Higher education: Pearson’s correlation coefficient = 0.47528, and the total population: Pearson’s correlation coefficient = 0.55871, respectively). A statistically significant correlation was observed between smoking cognition and positive behavior to quit smoking (Basic education: Pearson’s correlation coefficient = 0.42428, Secondary education: Pearson’s correlation coefficient = 0.19576, and the total population: Pearson’s correlation coefficient = 0.17406, respectively). However, no statistically significant correlation was observed between smoking cognition and positive behavior to quit smoking in a sample of higher education (Pearson’s correlation coefficient = 0.03012, *p* = 0.6881). Statistically significant correlation was observed between smoking attitude and positive behavior to quit smoking (Basic education: Pearson’s correlation coefficient = 0.44318, Secondary education: Pearson’s correlation coefficient = 0.30447, Higher education: Pearson’s correlation coefficient = 0.16996, and the total population: Pearson’s correlation coefficient = 0.27582, respectively). A statistically significant correlation was observed between smoking cognition, smoking attitude and positive behavior to quit smoking (Basic education: Pearson’s correlation coefficient = 0.46442, Secondary education: Pearson’s correlation coefficient = 0.26173, and the total population: Pearson’s correlation coefficient = 0.23272, respectively). However, no statistically significant correlation was observed between smoking cognition and positive behavior to quit smoking in a sample of higher education (Pearson’s correlation coefficient = 0.08869, *p* = 0.2364). Refer to Table 5.

**Table 5:** The correlation analysis of knowledge, attitude and behaviors, Chongqing, China, 2011.

Correlation	Basic Education (*n* = 67)		Secondary Education (*n* = 289)		Higher Education (*n* = 180)		The Total Population (*n* = 536)	
	Pearson Correlation Coefficient	*p*-Value	Pearson Correlation Coefficient	*p*-Value	Pearson Correlation Coefficient	*p*-Value	Pearson Correlation Coefficient	*p*-Value
Smoking cognition and attitude	0.68877	<0.0001 **	0.54064	<0.0001 **	0.47528	<0.0001 **	0.55871	<0.0001 **
Smoking cognition and behavior	0.42428	0.0003 **	0.19576	0.0008 **	0.03012	0.6881	0.17406	<0.0001 **
Smoking attitude and behavior	0.44318	0.0002 **	0.30447	<0.0001 **	0.16996	0.0226 *	0.27582	<0.0001 **
Smoking cognition, smoking attitude and behavior	0.46442	<0.0001 **	0.26173	<0.0001 **	0.08869	0.2364	0.23272	<0.0001 **

Notes: (1) * Statistically significant (*p* < 0.05); ** Statistically significant (*p* < 0.001); (2) The total score of smoking cognition is Table 2 all scores addition; (3) The total score of smoking attitude is Table 3 all scores addition; (4) The total score of smoking behavior (positive behavior to quit smoking) is Table 4 all scores addition, except the amount of smoking Compared with the past 1 month; (5) Smoking cognition, smoking attitude is the total score of “smoking cognition is Table 2 all scores addition” and “The total score of smoking attitude is Table 3 all scores addition”.

### 3.6. Factors Associated with Quitting Smoking Ideas

Several factors were considered in the modeling of quitting smoking idea among smokers: Demographic characteristics (age, education level); Cognition knowledge of smoking-related hazards (“Smoking cigarettes cause serious harm to one’s health”, “Smoking causes harm to my health”, “Smoking causes lung disease”, “Smoking causes oral cancer”, “Smoking causes heart diseases”, “Smoking causes stroke”, “Smoking causes Impotence”, “Exposure to second-smoke causes Lung cancer in non-smokers”, “Exposure to second-smoke causes Lung disease in children”, “Exposure to second-smoke causes Heart disease”, “Birth of low-weight babies when the pregnant mother has been exposed to cigarette smoking”); Smoking-related attitude (“The people around me (including family members, friends, and colleagues) believe that I should not smoke”, “Whether to support the provisions that establishment smoking bans in public places/workplace”, “Exposure to smoke from another person’s cigarette causes harm to my health”, “Quitting smoking would improve my health”); Smoking –related behavior (“The amount of smoking compared with the past 1 months”, “The people around me persuade me to quit smoking”). Logistic regression model to predict factors affect quitting smoking idea (Table 6), which indicates the factors affect quitting smoking ideas in this study. 

**Table 6:** Smoking-related knowledge, attitudes, behaviors and sociodemographic factors associated with quitting smoking idea Chongqing, China, 2011 (*n* = 536).

Predictors	Crude OR (95% CI)	Adjusted OR (95% CI)
*Demographic characteristics*		
Age		
26–35 years *vs.* 18–25years	1.304 (0.798, 2.131)	1.309 (0.785, 2.184)
36–45 years *vs.* 18–25years	1.732 (1.050, 2.858) *	1.755 (1.016, 3.031) *
Education level		
Secondary education *vs.* Basic education	---	1.077 (0.566, 2.046)
Higher education *vs.* Basic education	---	1.073 (0.520, 2.217)
*Cognition knowledge of smoking-related hazards*		
Smoking causes serious harm to one’s health		
Right *vs.* Wrong	0.580 (0.322, 1.046)	0.580 (0.321, 1.048)
Smoking causes harm to my health		
Right *vs.* Wrong	0.582 (0.353, 0.960) *	0.579 (0.350, 0.958) *
Smoking cause lung disease		
Right *vs.* Wrong	0.570 (0.227, 1.428)	0.563 (0.223, 1.421)
Smoking cause oral cancer		
Right *vs.* Wrong	1.178 (0.725, 1.916)	1.172 (0.716, 1.917)
Smoking cause heart diseases		
Right *vs.* Wrong	1.262 (0.734, 2.169)	1.261 (0.732, 2.173)
Smoking cause stroke		
Right *vs.* Wrong	0.569 (0.292, 1.106)	0.566 (0.290, 1.105)
Smoking cause Impotence		
Right *vs.* Wrong	1.817 (1.033, 3.196) *	1.812 (1.029, 3.191) *
Exposure to second-smoke cause Lung cancer in non-smokers		
Right *vs.* Wrong	1.355 (0.779, 2.358)	1.358 (0.780, 2.362)
Exposure to second-smoke cause Lung disease in children		
Right *vs.* Wrong	0.863 (0.479, 1.556)	0.860 (0.477, 1.552)
Exposure to second-smoke cause Heart disease		
Right *vs.* Wrong	0.704 (0.404, 1.226)	0.707 (0.405, 1.235)
Birth of low-weight babies when the pregnant mother has been exposed to cigarette smoking		
Right *vs.* Wrong	1.393 (0.838, 2.318)	1.388 (0.833, 2.313)
*Smoking-related attitude*		
The people around me (including family members, friends, and colleagues) believe that I should not smoke		
Disagree *vs.* Agree	0.567 (0.356, 0.904) *	0.568 (0.356, 0.905) *
Whether to support the provisions that establishment smoking bans in public places/workplace		
Disagree *vs.* Agree	0.924 (0.442, 1.933)	0.923 (0.441, 1.933)
Exposure to smoke from another person’s cigarette causes harm to my health		
Disagree *vs.* Agree	0.824 (0.500, 1.358)	0.825 (0.500, 1.360)
Quitting smoking would improve my health		
Disagree *vs.* Agree	0.609 (0.372, 0.997) *	0.610 (0.372, 1.000)
*Smoking-related behavior*		
The amount of smoking compared with the past 1 month		
Smoke Same *vs.* Smoke Less	0.195 (0.085, 0.446) *	0.194 (0.085, 0.444) *
Smoke More *vs.* Smoke Less	0.411 (0.199, 0.847) *	0.410 (0.199, 0.845) *
Hard to say *vs.* Smoke Less	0.505 (0.217, 1.172)	0.501 (0.215, 1.167)
The people around me persuade me to quit smoking		
No *vs.* Yes	0.483 (0.313, 0.744) *	0.482 (0.312, 0.743) *

Notes: (1) * There was statistically significant (*p* < 0.05); (2) Crude OR was unadjusted for education level; (3) Adjusted OR was adjusted for education level and all the variables in the table; (4) Abbreviation: CI: confidence intervals, OR: odds ratio.

Education level did not significantly predict have quit smoking idea. Compared with people of 18–25 years of age, people of 36–45 years of age (Adjusted OR = 1.755, 95% CI (1.016–3.031)) were more likely to have quit smoking. Male smokers who have the right cognition of “Smoking causes harm to my health” are less likely to have quitting smoking ideas (Adjusted OR = 0.579, 95% CI (0.350–0.958)). Male smokers who have right cognition of “Smoking causes impotence” were more likely to have quit smoking (Adjusted OR = 1.812, 95% CI (1.029–3.191)). Smokers who disagreed that the people around them believed that they should not smoke were less likely to have quit smoking ideas (Adjusted OR = 0.568, 95% CI (0.356–0.905)). The smokers who disagreed that quitting smoking would improve their health were less likely to have quitting smoking ideas (Adjusted OR = 0.610, 95% CI (0.372–1.000)). Compared with the past 1 month, participants who smoked same (OR = 0.194, 95% CI (0.085–0.444)) and smoked more (OR = 0.410, 95% CI (0.199–0.845)) than those who smoked less were less likely to have quit smoking. The smokers who believed that the people around them persuaded them to quit smoking, were inclined to answer “no” were less likely to have quit smoking ideas (Adjusted OR = 0.482, 95% CI (0.312–0.743)). Refer to Table 6.

## 4. Discussion

Our research showed that young adult males with higher education was more likely to have a better understanding of smoking being harmful to health, and showed more positive attitudes towards smoking-related hazards among young adult male smokers. These findings further confirm that awareness of the health hazards posed by smoking was correlated with education [21]. Previous studies showed that those with high levels of education and high socioeconomic status (SES) are more likely to be non-smokers [22,23]. Other research also showed that highly educated men have shown a decreasing smoking trend compared with the less educated groups in many European countries [24]. Possible reasons are that the people with better education might better understand health information, which could be better at translating health information into action, have a higher locus of control and may have more information and cognition about the importance of quitting smoking for disease management [22,25].

Our results further confirmed that smokers of different education levels have certain differences on smoking-related attitudes [26]. This survey revealed that male smokers’ attitude of smoking’s related hazards among high educational level was more positive than those of low education level. However, no significant difference was observed between the level of education and smoking-related attitudes in many aspects, such as “quitting smoking would improve my health” and “support the provisions that establish smoking bans in public places/workplace”. In this study, the smoking-related attitudes which were significant were all about the second-smoke, which is related to knowledge. So, these findings support the idea that level of education alone might have little impact on male smokers’ smoking-related attitudes.

Despite higher awareness of the disadvantages of smoking and a positive attitude towards quitting smoking, it did not necessarily affect some practices in a sample of young adult male smokers. Higher education has higher awareness of the disadvantages of smoking and a positive attitude towards smoking. Our results found that no statistically significant correlation was observed between smoking cognition and positive behavior to quit smoking in a sample of higher education, and no statistically significant correlation was observed between smoking cognition and positive behavior to quit smoking in a sample of higher education. Our results found that the smoking-related attitudes had little influence in practice in a sample of higher education. This result may be in agreement with a study at a Public and Islamic Azad University [1]. We guess the possible reasons include: smokers may have no perseverance and patience to put knowledge and attitude into action; degree of family intervention and supervision may be not enough. In addition to, increase provision of smoking cessation counseling by healthcare providers [21]. An education campaign accompanying the policy might be more effective in further reducing current cigarette use [27].

Logistic regression analysis indicated that seven factors were associated with quitting smoking idea in a sample of young adult male smokers. Respondents aged 36~45 years are more likely to have quit smoking ideas than respondents aged 18–25 years. Perhaps the most striking finding from our study was that education was not the factor that affects male smokers quit smoking ideas; previous research shows that women with more than 12 years of education were 27 times more likely than those with less than a high school education to have quit smoking [28]. This research’s population contains pregnant women, who are more likely to be persuaded to quit smoking [29], and our study only had male smokers, this was different from other study. There was another study which also showed that education was a significant moderator of smoking cessation upon diagnosis among adults ages 50–60, but not among adults ages 61–75 [22], but our study’s population were from age 18–45. This may be the reason why our results show that the education level doesn’t affect male smokers’ quitting smoking. Our study found that the people around me persuaded me to quit smoking, smokers were inclined to answer “no” were less likely to have quit smoking ideas. This further confirmed that people who were easier to quit smoking were having people around them to persuade them [30]. This indicated that the power from people around smokers, such as friends, parents, brothers and sisters cannot be overlooked for quitting smoking. The amount of smoking was an important factor associated with quitting smoking ideas. Participants who smoked the same and smoked more than those who smoked less were less likely to have quit smoking. Smokers who disagreed that quitting smoking would improve my health are less likely to have quit smoking. The current study dealt with limiting factors of knowledge, attitude and the behavior, some other factors also affect the quitting smoking idea. Numerous studies have investigated many other factors (spouse and friends’ smoking, had an influence on affect cessation). Having children in the home might be expected to increase motivation to quit [31]. Living with smokers has a negative impact on cessation [32]. Smoking dependence and household smoking bans also have an influence on cessation [33]. Health education workers need to fully consider these factors in future planning for helping smokers quit smoking. 

This study also had certain limitations. First, the use of cross-sectional survey data reduced the researchers’ ability to make direct causal inferences, to explore whether unmeasured factors may better explain the observed relationships we observed, and determine the direction of causality. Second, our sample may not be representative of all young male smokers, representativeness of the sample was carried out from the research methods was insufficient. We did not take the probability sampling. Third, our study only investigates the city dweller, no investigation of rural male smokers. China was the world’s most populous country with 1.3 billion people and more than half of them live in the rural areas [34]. Smoking prevalence was higher among rural residents [19] and particularly among adult males [35]. It would be helpful to replicate our work in other settings. Fourth, this study did not assess the concurrent validity of the questionnaire. This questionnaire was our own design. We did not assess the validity of the questionnaire with measures that did not themselves have established validity limits the validity of the findings. What’s more, this study conducted in China, because of the different social system and culture, the education was different in different countries. Further studies are needed in other countries or regions to further confirm and improve the applicability of the conclusion.

## 5. Conclusions

In conclusion, these findings support the idea that young adult males with higher education had a more positive attitude toward smoking, however, this knowledge and attitude did not necessarily translate into health behavioral outcomes. There was no statistically significant correlation between smoking cognition and smoking behavior among young male smokers in a sample with higher education, and also no statistically significant correlation was observed between smoking cognition and smoking behavior in a sample with higher education. Our study indicated that education level might be not a factor that affect think about quitting smoking ideas among young male smokers in China. These findings provided insight into health education in tobacco control in transition. This survey gave a snapshot of the impact of education on young adult male smokers’ idea of quitting smoking and prospective studies on this topic are warranted to confirm findings from the present study. This study also allowed us to think the current education in China, and education should pay more attention to health education. In the future of tobacco control research, especially for smokers’ study, not only we should pay attention to the low educational level of individuals, but also to the high education level of smokers.

## References

[1] Askarian M., Kouchak F., Youssef M., Romito L.M. (2013). Romito comparing tobacco use knowledge, attitudes and practices between engineering students at a public and Islamic Azad university in Shiraz, Iran 2011. Int. J. Prev. Med..

[2] Mathers C.D., Loncar D. (2006). Projections of global mortality and burden of disease from 2002 to 2030. Public Libr. Sci. Med..

[3] Hammond D., Fong G.T., McNeill A., Borland R., Cummings K.M. (2006). Effectiveness of cigarette warning labels in informing smokers about the risks of smoking: Findings from the International Tobacco Control (ITC) four country survey. Tob. Control.

[4] National Center for Chronic Disease Prevention and Health Promotion, U.S. Office on Smoking and Health (2014). The Health Consequences of Smoking—50 Years of Progress: A Report of the Surgeon General.

[5] Guindon G.E., Boisclair D. (2003). Past, Current and Future Trends in Tobacco Use: HNP Discussion Paper.

[6] (2013). WHO Report on the Global Tobacco Epidemic, Country Profile China, 2013: The MPOWER Package.

[7] (2009). Global Adult Tobacco Survey: Egypt Country Report 2009/World Health Organization.

[8] Hermalin A.I., Lowry D. (2010). The Age Prevalence of Smoking among Chinese Women: A Case of Arrested Diffusion.

[9] Geckova A., Dijk J.P., Groothoff J.W., Post D. (2002). Socio-economic differences in health risk behaviour and attitudes towards health risk behaviour among Slovak adolescents. Soz. Praventivmed..

[10] Ilhan I.O., Yildirim F., Demirbas H., Dogan Y.B. (2009). Prevalence and sociodemographic correlates of substance use in a university-student sample in Turkey. Int. J. Public Health.

[11] Piko B.F., Fitzpatrick K.M. (2007). Socioeconomic status, psychosocial health and health behaviours among Hungarian adolescents. Eur. J. Public Health.

[12] Warren C.W., Jones N.R., Eriksen M.P., Asma S. (2006). Patterns of global tobacco use in young people and implications for future chronic disease burden in adults. Lancet.

[13] Feng G., Jiang Y., Li X., Yang Y., Wu X. (2009). Survey on smoking-related knowledge, attitudes and behavior of teachers across China. Chin. J. Prev. Control Chronic Dis..

[14] Smit E.S., Fidler J.A., West R. (2011). The role of desire, duty and intention in predicting attempts to quit smoking. Addiction.

[15] Mirowsky J., Ross C.E. (1998). Education, personal control, lifestyle and health: A human capital hypothesis. Res. Aging.

[16] Backlund E., Sorlie P.D., Johnson N.J. (1999). A comparison of the relationships of education and income with mortality: The national longitudinal mortality study. Soc. Sci. Med..

[17] Siahpush M., McNeill A., Hammond D., Fong G.T. (2006). Socioeconomic and country variations in knowledge of health risks of tobacco smoking and toxic constituents of smoke: Results from the 2002 International Tobacco Control (ITC) four country survey. Tob. Control.

[18] Al Riyami A.A., Afifi M. (2004). Smoking in Oman: Prevalence and characteristics of smokers. East. Mediterr. Health J..

[19] Chinese Center for Disease Control and Prevention (2011). Global Adult Tobacco Survey (GATS) China Fact Sheet 2010.

[20] Yang G.H., Ma J.M., Liu N., Zhou L.N. (2005). Smoking and passive smoking in Chinese, 2002. Zhonghua Liu Xing Bing Xue Za Zhi.

[21] Demaio A.R., Nehme J., Otgontuya D., Meyrowitsch D.W., Enkhtuya P. (2014). Tobacco smoking in Mongolia: Findings of a national knowledge, attitudes and practices study. BMC Public Health.

[22] Margolis R. (2013). Educational differences in healthy behavior changes and adherence among middle-aged Americans. J. Health Soc. Behav..

[23] Benson F.E., Stronks K., Willemsen M.C., Bogaerts N.M.M., Nierkens V. (2014). Wanting to attend isn’t just wanting to quit: Why some disadvantaged smokers regularly attend smoking cessation behavioural therapy while others do not: A qualitative study. BMC Public Health.

[24] Giskes K., Kunst A.E., Benach J., Borrell C., Costa G., Dahl E., Dalstra J.A., Federico B., Helmert U., Judge K. (2005). Trends in smoking behaviour between 1985 and 2000 in nine European countries by education. J. Epidemiol. Community Health.

[25] Ockene J.K., Mermelstein R.J., Bonollo D.S., Emmons K.M., Perkins K.A., Voorhees C.C., Hollis J.F. (2000). Relapse and maintenance issues for smoking cessation. Health Psychol..

[26] Lim K.H., Sumarni M.G., Amal N.M., Hanjeet K., Wan Rozita W.M., Norhamimah A. (2009). Tobacco use, knowledge and attitude among Malaysians age 18 and above. Trop. Biomed..

[27] Chaaya M., Alameddine M., Nakkash R., Afifi R.A., Khalil J., Nahhas G. (2013). Students’ attitude and smoking behaviour following the implementation of a university smoke-free policy: A cross-sectional study. BMJ Open.

[28] Higgins S.T., Heil S.H., Badger G.J., Skelly J.M., Solomon L.J., Bernstein I.M. (2009). Educational disadvantage and cigarette smoking during pregnancy. Drug Alcohol Depend..

[29] Bennett L., Grant A., Jones S., Bowley M., Heathcote-Elliott C., Ford C., Jones A., Lewis R., Munkley M., Owen C. (2014). Models for Access to Maternal Smoking cessation Support (MAMSS): A study protocol of a quasi-experiment to increase the engagement of pregnant women who smoke in NHS stop smoking services. BMC Public Health.

[30] Rees V.W., Keske R.R., Blaine K., Aronstein D., Gandelman E., Lora V., Savage C., Geller A.C. (2014). Factors influencing adoption of and adherence to indoor smoking bans among health disparity communities. Amer. J. Public Health.

[31] Borland R., Yong H.H., Cummings K.M., Hyland A., Anderson S., Fong G.T. (2006). Determinants and consequences of smoke-free homes: Findings from the International Tobacco Control (ITC) four country survey. Tob. Control.

[32] Osler M., Prescott E. (1998). Psychosocial, behavioural, and health determinants of successful smoking cessation: A longitudinal study of Danish adults. Tob. Control.

[33] Biener L., Hamilton W.L., Siegel M., Sullivan E.M. (2010). Individual, social-normative, and policy predictors of smoking cessation: A multilevel longitudinal analysis. Amer. J. Public Health.

[34] National Bureau of Statistics of China (2009). China Statistical Yearbook 2009.

[35] Li Q., Hsia J., Yang G. (2011). Prevalence of Smoking in China in 2010. N. Engl. J. Med..

